# Editorial: Glial Dysfunction in Epileptogenesis

**DOI:** 10.3389/fneur.2021.716308

**Published:** 2021-07-13

**Authors:** Kjell Heuser, Marco de Curtis, Christian Steinhäuser

**Affiliations:** ^1^Department of Neurology, Division of Clinical Neuroscience, Oslo University Hospital, Rikshospitalet, Oslo, Norway; ^2^Epilepsy Unit, Fondazione Istituto di Ricovero e Cura a Carattere Scientifico (IRCCS) Istituto Neurologico Carlo Besta, Milan, Italy; ^3^Institute of Cellular Neurosciences, Medical Faculty, University of Bonn, Bonn, Germany

**Keywords:** epilepsy, glia, epileptogenesis, temporal lobe epilepsy, astrocyte

Epilepsy is one of the most common neurological disorders affecting around 1% of the world's population (1). Epilepsy research has so far mainly focused on how to inhibit epileptic discharges resulting in different symptomatic treatment options targeted at neurons.

Despite the introduction of more than 20 novel anti-seizure medications since the early 1990s, the proportion of people with drug-refractory epilepsy has remained notably stable at ~30% (2).

Given the expanding spectrum of important functions ascribed to the non-neuronal constituents of the brain, we can now observe a paradigm shift where glial cells are included in the equation of epilepsy pathogenesis. With our novel understanding of glial cells as central organizers of homeostatic functions and as major contributors to inflammation and brain excitability, we are approaching novel curative treatment strategies for epilepsy. In the future, targeting dysfunctional and/or reactive glia or glia-mediated inflammatory processes may thus prevent initiation and progression of epilepsy, and yield true anti-epileptogenic medications.

In this special issue (SI) of Frontiers in Neurology, we brought together experts in this new area of epilepsy research, and provide a balanced collection of seven original studies and eight review articles.

The eBook both starts and ends with reviews of one of the core homeostatic functions of astrocytes, which is glutamate handling in the central nervous system. Glutamate clearance is highly relevant for epilepsy pathogenesis as excess glutamate directly could trigger neuronal discharges and epileptic activity. The first article, presented by Peterson and Binder (USA), provides a systematic overview of the functional components of astrocyte glutamate control. Astrocytes express both glutamate transporters (GLT-1 and GLAST in rodents/EAAT 1 and 2 in humans), as well as metabotropic glutamate receptors (mGluR)3 and mGluR5. Peterson and Binder demonstrate evidence for dysregulation of these channels across patients with epilepsy and preclinical seizure models.

After uptake, astrocytes conduct the intracellular metabolization of glutamate and ammonia to glutamine. Sandhu et al. from the group of Tore Eid (USA) highlight the importance of the enzyme glutamine synthetase (GS), and provide evidence for the association of astrocytic GS deficiency or dysfunction in discrete brain regions in several types of epilepsy, including mesial temporal lobe epilepsy, neocortical epilepsies, and glioblastoma-associated epilepsy. These findings are reinforced by several studies using experimental inhibition or deletion of GS in specific brain regions, and by this mimic different human epilepsy forms including their comorbidities.

Alcoreza et al. from the group of Harald Sontheimer (USA) provide information on astrocytic glutamate receptor dysregulation in epilepsy, presenting yet another view of the delicate regulatory processes of glutamate homeostasis. They also highlight the importance of extracellular space volume alterations and dysregulation of the water channel aquaporin-4 as integral parts of epilepsy pathophysiology and discuss evidence for upregulation of system x–c, a cystine/glutamate antiporter expressed by astrocytes in epileptic tissue.

Kinboshi et al. (Japan) focuses on another important homeostatic function of astrocytes, which is K^+^ spatial buffering. This process mainly depends on inwardly rectifying potassium (Kir) 4.1 channels and gap junction coupling. Astrocytes rapidly transport K^+^ from areas of high neuronal activity, where [K^+^]_EC_ increases, to regions with lower K^+^ levels via the astrocyte network through gap junctions. This K^+^ clearance mechanism is not only critical for maintaining K^+^ homeostasis and preventing neural hyperexcitability but is also linked to glutamate uptake during normal brain function. There is ample evidence for Kir4.1 dysfunction in epilepsy and recent studies indicate that inhibition of Kir4.1 channels facilitates the expression of brain-derived neurotrophic factor (BDNF), an important modulator of epileptogenesis in astrocytes.

Verhoog et al. from the group of Erwin van Vliet (The Netherlands) enriches this eBook with a substantial review of state-of-the-art literature on dysfunctional astrocytes in epilepsy, also including the role astrocyte Ca^2+^ signaling, altered blood brain barrier (BBB) function and blood flow regulation.

The series of contributions to this eBook on glia-derived inflammation in epilepsy could not have a better opening than with an original study on febrile seizures, provided by Brennan et al. from the group of Tallie Baram (USA). The potential role of aberrant microglia and astrocyte function during epileptogenesis is important because the involved mediators provide targets for intervention and prevention of epilepsy. By performing experimental febrile status epilepticus in rat pups, the authors elicited a strong inflammatory response leading to a rapid and sustained upregulation of pro-inflammatory cytokines. In the attempt to curb epileptogenesis, several pathways involving cytokines, microRNAs, high mobility group B-1 (HMGB1) and prostaglandin E2 signaling were targeted by using network-specific interventions as well as global anti-inflammatory approaches. The failure of selectively decreasing the expression of downstream inflammatory cascades, and the emergence of intolerable side effects, illustrates that the intricate, cell-specific and homeostatic interplays among these networks constitute a serious challenge to tailored interventions that aim to prevent epileptogenesis.

Another approach to curb epileptogenesis is presented in the study of Wyatt-Johnson et al. (USA). Microglial survival and proliferation are regulated by the colony-stimulating factor 1 receptor (CSF1R). The authors used the CSF1R inhibitor PLX3397 in a rat pilocarpine model of status epilepticus (SE). This led to suppression of microgliosis and hippocampal astrogliosis but did not improve or worsen the memory deficits in these animals.

Neuroinflammation is not only an integral part of TLE pathogenesis, but also regarded as a hallmark of traumatic brain injury (TBI) and subsequent post-traumatic epilepsy (PTE). Sun et al. (China) presents an organized review focusing particularly on glial cell activation, peripheral leukocyte infiltration, inflammatory cytokine release and BBB disruption in PTE.

BBB disruption is a hallmark of many pathological brain insults and has been associated with the development and progression of focal epilepsy, although the underlying molecular mechanisms are not fully understood. A proposed mechanism is the activation of transforming growth factor beta (TGFβ) signaling in astrocytes by extravasated albumin, impairing the ability of astrocytes to properly interact with neurons and eventually leading to epileptiform activity. Henning et al. (Germany) used the unilateral intracortical kainate mouse model of TLE with hippocampal sclerosis (HS) and revealed pronounced albumin extravasation already 4 h after SE induction. Inhibition of the TGFβ pathway by the specific TGFβ receptor 1 (TGFβR1) kinase inhibitor IPW-5371 slightly attenuated acute and chronic epileptiform activity but did not reduce the extent of HS or affect astrocytic gap junction coupling, which is thought to play a role in TLE-HS epileptogenesis (3). The same group, with Müller et al. (Germany) as first author, presents another original study employing the same mouse model. Accompanied by loss of GABAergic interneurons and/or synaptic inhibition, as shown in various epilepsy models and in human epilepsy, they found a pronounced GABA accumulation in reactive astrocytes of the sclerotic mouse hippocampus.

Together, their data provide evidence that the preserved tonic inhibitory currents in the epileptic brain are mediated by GABA overproduction and release from astrocytes, adding another potential target for antiepileptogenic drug therapy.

Vila Verde et al. (Italy) contribute with an elegant approach using the guinea pig epilepsy model (4) and, for the first time, provide evidence that seizures *per se* induce IL-1β biosynthesis in astrocytes, increased BBB permeability, and morphological changes typically observed in activated glial cells, in the absence of blood borne inflammatory molecules and leukocytes. They further found that serum albumin extravasation into the brain parenchyma exacerbates neuronal hyperexcitability by inducing astrocytic and microglial activation.

Ahmed et al. from the group of Brooks-Kayal (USA) provide one out of 2 “omics”-contributions investigating upstream effects of epileptogenesis. Using a mouse pilocarpine TLE model they utilized the moderate throughput technique of Reverse Phase Protein Arrays (RPPA) and measured levels of proteins comprising components of major signaling pathways and cellular complexes and found time- and region-specific changes in correlations among levels of functionally related proteins affecting both neurons and glia. Among these they identified changes of levels of the MTOR pathway component pS6, and detailed responses of multiple components of the MTOR, MAPK, JAK/STAT and apoptosis pathways, NMDA receptors, and additional cellular complexes.

In the other “omics”- approach, also relatable to the Vila Verde et al. study presented earlier exploring the effects of seizure activity *per se*, Berger et al. (Norway) investigated upstream-effects of early epileptogenesis in the contralateral hippocampus (CLH) of mice treated with the intracortical kainate model of TLE with HS. They found that the CLH, despite the absence of morphological changes, shows substantial changes in gene expression and DNA methylation in both glia and neurons, but displays a significantly lower number of glial genes up- and downregulated compared to earlier results from the ipsilateral hippocampus (5). Furthermore, several genes and pathways potentially involved in “anti-epileptogenic effects” were upregulated in the CLH, suggesting compensatory mechanisms to prevent morphological alterations like neuronal death and reactive gliosis.

While most contributions to this eBook have focused on glial dysfunction during epileptogenesis in TLE with HS, two contributions broach the issue of other epilepsy conditions. The first one is a review on Tuberous sclerosis complex (TSC) by Zimmer et al. from the group of Elenora Aronica (The Netherlands). TSC represents the prototypic monogenic disorder of the mammalian target of rapamycin (mTOR) pathway dysregulation and is associated with structural and functional brain abnormalities and intellectual disability. So far, research conducted in TSC has been largely neuron-centered. This review highlights recent achievements in TSC research focusing on glial cells, which now are believed to be integral parts of the pathological features of this condition. These cells and their inter-glial crosstalk may offer new insights into the common neurobiological mechanisms underlying epilepsy and the complex cognitive and behavioral comorbidities that are characteristic of the spectrum of mTOR-associated neurodevelopmental disorders.

The last contribution is a review by Gobbo et al. (Germany). The authors provide a substantial overview of the physiology and pathology of cortico-thalamo-cortical oscillations. The electrographic hallmark of childhood absence epilepsy and other idiopathic forms of epilepsy are 2.5–4 Hz spike and wave discharges (SWDs) originating from abnormal electrical oscillations of the cortico-thalamo-cortical network. SWDs are generally associated with sudden and brief non-convulsive epileptic events mostly generating impairment of consciousness and correlating with attention and learning as well as cognitive deficits. The authors deep-dig into this topic and provide substantial information on the role of astroglia (including interstitial fluid homeostasis, K^+^ clearance, neurotransmitter uptake, gap junction function, gliotransmission, astroglial Ca^2+^ signaling, and finally reactive astrogliosis and cytokine release) in the modulation of excitation and inhibition in the brain as well as on the development of aberrant synchronous network activity, also bridging over to sleep disturbances.

## Conclusion

In conclusion, this eBook embraces the complex and multifaceted contributions of glia function and dysfunction to epileptogenesis and illustrates in various ways the intricate interplay between glia and neurons in the etiology and pathogenesis of the epilepsies. Today, research on glia in epilepsy is still in its infancy. We allow us to postulate that increased research focus on glia in combination with novel technology represents an opportunity to develop therapeutic niches, including disease-modifying treatments and true anti-epileptogenic drugs (6).

## Author Contributions

KH, MdC, and CS wrote the eBook. All authors contributed and validated the Editorial.

## Conflict of Interest

The authors declare that the research was conducted in the absence of any commercial or financial relationships that could be construed as a potential conflict of interest.
